# Feasibility of Direct Sputum Molecular Testing for Drug Resistance as Part of Tuberculosis Clinical Trials Eligibility Screening

**DOI:** 10.3390/diagnostics9020056

**Published:** 2019-05-30

**Authors:** Narges Alipanah, Priya B. Shete, Hanh Nguyen, Nhung Viet Nguyen, Lien Luu, Thuong Pham, Hung Nguyen, Phuong Nguyen, Minh Chi Tran, Nam Pham, Ha Phan, Patrick P.J. Phillips, Adithya Cattamanchi, Payam Nahid

**Affiliations:** 1 Santa Clara Valley Medical Center, San Jose, CA 95128, USA; narges.alipanah@ucsf.edu; 2 UCSF Center for Tuberculosis, San Francisco, CA 94110, USA; Priya.Shete@ucsf.edu (P.B.S.); Patrick.Phillips@ucsf.edu (P.P.J.P.); Adithya.Cattamanchi@ucsf.edu (A.C.); 3 University of California, San Francisco, CA 94143, USA; steinriver@yahoo.com; 4 Vietnam National Tuberculosis Programme/UCSF Research Collaboration, Hanoi, Vietnam; hanh202000@gmail.com (H.N.); vietnhung@yahoo.com (N.V.N.); hungmtb75@gmail.com (H.N.); thanhnam.bvphn@gmail.com (N.P.); 5 National Reference Laboratory, National Lung Hospital, Hanoi, Vietnam; phuong125a@gmail.com; 6 Hanoi Lung Hospital, Hanoi, Vietnam; honglien38@gmail.com (L.L.); drthuongbvphn@gmail.com (T.P.); 7 Stanford Healthcare ValleyCare, Pleasanton, CA 94588, USA; minhchi.tran@gmail.com

**Keywords:** MTBDR*plus*, MTBDR*sl*, line probe assay, tuberculosis, DR-TB, Hain test, diagnostics, clinical trials, molecular testing

## Abstract

A rapid diagnosis of drug-resistant tuberculosis (TB) is critical for early initiation of effective therapy. Molecular testing with line probe assays (MTBDR*plus* and MTBDR*sl*) on culture isolates has been available for some time and significantly reduces the time to diagnosis of drug resistance. However, routine use of this test directly on sputum is less common. As part of enrollment screening procedures for tuberculosis clinical trials conducted in Hanoi, Vietnam, we evaluated the feasibility and performance of line probe assay (LPA) testing directly on sputum samples from 315 participants with no prior history of TB treatment. Test performance characteristics for the detection of rifampin (RIF) and isoniazid (INH) drug resistance as compared to culture-based drug susceptibility testing (DST) reference standard were calculated. LPA demonstrated high sensitivity and specificity for the diagnosis of drug resistance. Scaling up molecular testing on sputum as part of time-sensitive clinical trial screening procedures in high TB burden settings is feasible and will reduce both time to initiation of appropriate therapy and the risk of late exclusions due to microbiologic ineligibility.

## 1. Introduction

Tuberculosis (TB) remains a public health threat with an estimated 10 million people diagnosed and 1.3 million deaths worldwide in 2017 alone [1]. The global prevalence of drug-resistant tuberculosis (DR-TB) is increasing, yet only 25% of those newly infected with TB in 2017 were enrolled in treatment [1]. In response, the latest report by the World Health Organization highlights improvement in TB diagnostics and shortening the time to initiation of appropriate treatment as key priorities [1]. Furthermore, the recent guidelines recommend drug sensitivity testing in all settings, shifting the paradigm of TB treatment from standardized regimens to individualized therapy [2]. The identification of drug resistance by culture-based methods takes 8–12 weeks, delaying identification of individuals with DR-TB by several months. Though rapid molecular tests have been developed to identify DR-TB, questions surrounding their feasibility has limited their use in high TB burden settings. Moreover, the GeneXpert MTB/RIF assay, the most commonly accessible rapid molecular assay in the field, currently only tests for resistance to rifampin. In many program settings, additional information on isoniazid and fluoroquinolone resistance can be informative in selecting the appropriate combination regimen for the patient. In the context of clinical trial screenings, rapid detection of resistance to rifampin, isoniazid, and fluoroquinolones is essential for identifying eligible study participants. Rapid screening can prevent treating participants with drugs to which their bacteria have resistance, expedites access to curative therapy, and reduces risks for late exclusions, which impact the integrity of the clinical trial.

Molecular testing of culture isolates for drug resistance using commercial line probe assays (Hain Genotype^®^, Hain Lifesciences, Germany) has been extensively reported. In Vietnam, sensitivity of 93.1% for rifampin (RIF) resistance, 92.6% for isoniazid (INH) resistance, and 88.9% for multidrug-resistant TB (MDR-TB) have been reported with 100% specificity [3]. Although less commonly used, MTBDR*plus* tested directly on sputum samples has demonstrated acceptable test performance characteristics compared to culture when used on smear-positive sputum samples in several settings [4,5,6,7]. Nevertheless, molecular testing on sputum is not routinely conducted. Current practice across clinical trials networks and sites varies in terms of screening procedures and the use of rapid diagnostic testing for the detection of drug resistance. Three recent phase 3 trials for drug-sensitive TB (DS-TB) reported 6–12% of those enrolled as late exclusions due to drug resistance [8,9,10]. We sought to assess the feasibility and test characteristics of using line probe assay (LPA) testing directly on sputum as part of the screening for TB clinical trial enrollment in Hanoi, Vietnam.

## 2. Materials and Methods

This cross-sectional study was conducted during a screening of 394 participants for enrollment into CDC-TB Trials Consortium Study 29X (S29X) and Study 31 (S31), conducted in Hanoi, Vietnam, between February 2012 and October 2018. S29X (ClinicalTrials.gov Identifier: NCT00694629) and S31 (ClinicalTrials.gov Identifier: NCT02410772) were phase 2 and phase 3 clinical trials, respectively, each evaluating rifapentine (RPT)-based regimens for the treatment for DS-TB [11]. A clinical research nurse coordinator conducted standard enrollment procedures (informed consent, interview, chest X-ray, laboratory testing, sputum culture (Lowenstein–Jensen, LJ), and drug susceptibility testing (DST)) for all participants. In addition, the Genotype MTBDR*plus* VER 2.0 and MTBDR*sl* VER 2.0 assays (Hain Lifescience GmbH, Nehren, Germany) were performed directly on sputum specimens within 24–48 h of collection. MTBDR*plus* was used to detect resistance to rifampin (mutations in the *rpoB* gene) and isoniazid (*katG* and *inhA* genes), and MTBDR*sl* (Hain Lifescience GmbH, Nehren, Germany) was used to detect fluoroquinolones (*gyrA* gene) resistance. We followed the manufacturer’s directions to perform the assays. Results of resistance identified through molecular testing initiated a return evaluation, prerandomization exclusion from enrollment, and referral to the Vietnam National TB Programme (NTP) for further management of drug-resistant TB.

We calculated the sensitivity, specificity, positive predictive value (PPV), and negative predictive value (NPV) of line probe assay (LPA) using results of drug susceptibility testing (DST) on Lowenstein–Jensen (LJ) medium and the BACTEC Mycobacterial Growth Indicator Tube (MGIT; Becton Dickinson and Co., Franklin Lakes, NJ, USA) liquid medium with the MGIT 960 system as the reference standards. Drug-resistant TB (DR-TB) was defined as TB resistant to any of the first-line TB treatment agents. Multidrug-resistant TB (MDR-TB) was defined as TB resistant to isoniazid and rifampin. Statistical analysis was conducted using Excel (Microsoft, Redmond, WA, USA) and Stata 13 (StataCorp, College Station, TX, USA).

## 3. Results

A total of 394 participants were screened for enrollment in S29X and S31. Of these, 79 participants were excluded from analyses because of missing reference culture-based DST results, missing LPA results, indeterminate LPA results, negative culture for mycobacterium TB, or negative smears. Three hundred fifteen smear-positive participants with complete phenotypic and molecular assay results were included in the quantitative analysis. The study cohort had a median age of 40 years (range 17–79) and was 80% male. At the time of presentation, 74% of the participants had cavitary lesions on chest radiographs. The median sputum mycobacterial load was 2+ (interquartile range 1).

Phenotypic DST demonstrated 76 cases of INH monoresistance (24%), three cases of RIF monoresistance (0.9%), and MDR-TB in ten participants (3.2%). LPA identified 76/86 participants with INH-resistant TB (including MDR-TB) by phenotypic DST (sensitivity 88%, 95% Confidence Interval (CI) 80–94%) and was positive for INH resistance in six participants with INH-susceptible TB by phenotypic DST (specificity 97%, 95% CI 94–99%) (Table 1). 86% of participants identified by LPA with INH resistance harbored isolates with *katG* mutations. LPA identified 9/13 participants with rifampin-resistant TB (sensitivity 69%, 95% CI 39–91%) and 301/302 patients with rifampin-susceptible TB (specificity 100%, 95% CI 97–100%). LPA also identified 5/6 patients with ofloxacin-resistant TB (sensitivity 83%, 95% CI 36–100%) and 183/188 patients with ofloxacin-sensitive TB (specificity 97%, 95% CI 94–99%). LPA results were available to clinicians within a median of 31 h (interquartile range 25 h), enabling all 80 (27%) participants with positive molecular results for INH resistance to be excluded from the clinical trial prior to randomization and to be referred for initiation of appropriate therapies from the Vietnam NTP. The median time to treatment initiation in our study from the time of sputum collection was two days (interquartile range 2 days).

## 4. Discussion

Our experience in applying the Hain LPA directly on sputum for diagnosis of drug-resistant tuberculosis in Hanoi, Vietnam, supports its feasibility as a component of the screening process for TB clinical trials. Diagnostic performance characteristics for LPA in our context were similar to those reported in nontrial settings on direct sputum, with a sensitivity of 88% and a specificity of 97% for the detection of INH resistance. Sensitivity for RIF resistance was 69%, lower than reported elsewhere [6,7], and might be due to the low prevalence of RIF resistance in our study (only 13 patients), the existence of alternative mutations conferring rifampicin resistance than identified by LPA testing, or possible coexisting drug-resistant and drug-susceptible strains or heteroresistance [12].

The use of molecular testing as a screening tool enabled us to exclude one-third of the screened participants who would have otherwise been potentially randomized and enrolled into the trials, which consequently would have resulted in them receiving treatments to which their isolates were not fully susceptible. Of note, all screened participants reported no prior history of tuberculosis treatment, and the local epidemiology of drug resistance in different settings would impact the added value of routinely integrating rapid molecular testing for participants screened for clinical trials. The prevalence of INH resistance in our study population was 27%, which was higher than the 16–20% prevalence previously reported in programmatic data from Vietnam [13]. Early detection of drug resistance in DR-TB endemic settings such as ours could have a significant impact on the number of participants excluded from clinical trials due to microbiological ineligibility, making studies more efficient and less costly, as well as assuring with greater certainty that patients infected with isolates with drug resistance are initiated on appropriate therapies. Indeed, early detection of INH resistance by MTBDR*plus* enabled all 82 participants with INH resistance identified by MTBDR*plus* (including nine participants with MDR-TB) to be promptly referred to the Vietnam NTP for treatment initiation using appropriate drug regimens. In light of rising rates of drug resistance globally and the risks of acquisition of additional resistance when inappropriate regimens are used, scaling up molecular testing on sputum would allow for more timely initiation of effective therapy. Indeed, the feasibility of rapid molecular testing on sputum samples has been associated with a median decrease in time of 25–66 days to appropriate treatment initiation in some programmatic settings [14,15]. Rapid molecular testing diagnostic algorithms have also been shown to correspond to as much as 4.5 times reduction in healthcare cost per patient when compared to phenotypic DST [16].

Our study has several limitations. Our experience applies to Hanoi, Vietnam, and the performance characteristics of LPA testing may differ in other clinical trial contexts with higher or lower endemic levels of drug resistance. Although LPA testing in our study provided a result within 36 h after the screening visit, requiring close collaboration between field and laboratory personnel, this might not be possible in all settings where infrastructure and expertise for line probe assay testing are limited. Nonetheless, our results underscore the feasibility of this approach to rapid molecular testing for drug resistance that could be applied in programmatic settings that have the appropriate support as our clinical trial unit which is embedded in the Vietnam NTP.

Early and rapid detection of drug resistance in the clinical trial setting has several potential benefits such as improved efficiency, cost, and patient care that will allow trials to perform in keeping with good clinical practices. In addition, the use of molecular drug susceptibility testing in clinical trials will reduce the risk of late exclusions due to microbiologic ineligibility. Routine adoption of molecular assays applied directly to sputum for the detection of drug resistance is an effective means of improving current TB clinical trials practice. Beyond the clinical trial setting, the scale-up of molecular testing on sputum will assure that the appropriate therapy is initiated for patients with drug resistance and that TB treatment outcomes are improved.

## Figures and Tables

**Table 1:** Performance of MTBDR*plus* testing on sputum samples compared with culture-based drug susceptibility testing (DST) results. INH = isoniazid. MDR-TB = multidrug-resistant TB. NPV = negative predictive value. OFX = ofloxacin/moxifloxacin. PPV = positive predictive value. RIF = rifampin.

	INH		RIF		OFX		MDR-TB	
	%	95% CI	%	95% CI	%	95% CI	%	95% CI
Sensitivity	88	80–94	69	39–91	83	36–100	80	44–97
Specificity	97	94–99	100	98–100	97	94–99	100	98–100
PPV	93	85–97	90	55–100	50	19–81	89	52–100
NPV	96	92–98	99	97–100	99	97–100	99	98–100
